# Quantitative analysis of endobronchial elastography combined with serum tumour markers of lung cancer in the diagnosis of benign and malignant mediastinal and hilar lymph nodes

**DOI:** 10.3389/pore.2023.1611377

**Published:** 2023-11-30

**Authors:** Zhen Wang, Peng Li, Jiayu Bai, Yujia Liu, Guangyu Jiao

**Affiliations:** ^1^ Department of Respiratory Medicine, Shengjing Hospital of China Medical University, Shenyang, China; ^2^ Department of Rheumatology, The First Affiliated Hospital of China Medical University, Shenyang, China; ^3^ College of Traditional Chinese Medicine, Liaoning University of Traditional Chinese Medicine, Shenyang, China

**Keywords:** elastography, endobronchial ultrasound-guided transbronchial needle aspiration, mediastinal and hilar lymph nodes, lung cancer, serum tumour markers

## Abstract

**Purpose:** In malignant tumours, elastography and serum tumour markers have shown high diagnostic efficacy. Therefore, we aimed to quantitatively analyse the results of endobronchial elastography combined with serum tumour markers of lung cancer to accurately distinguish benign and malignant mediastinal and hilar lymph nodes.

**Methods:** Data of patients who underwent endobronchial ultrasound-guided transbronchial needle aspiration for mediastinal lymph node enlargement in our hospital between January 2018 and August 2022 were retrospectively collected. The characteristics of quantitative elastography and serum tumour markers were evaluated.

**Results:** We enrolled 197 patients (273 lymph nodes). In the differential diagnosis of benign and malignant mediastinal and hilar lymph nodes, the stiffness area ratio (SAR), strain ratio (SR), and strain rate in lymph nodes were significant, among which SAR had the highest diagnostic value (cut-off value, 0.409). The combination of the four tumour markers had a high diagnostic value (AUC, 0.886). Three types of quantitative elastography indices combined with serum tumour markers for lung cancer showed a higher diagnostic value (AUC, 0.930; sensitivity, 83.5%; specificity, 89.3%; positive predictive value, 88.1%; negative predictive value, 85%) (*p* < 0.05). In the differential diagnosis of pathological types of lung cancer, different quantitative elastography indicators and serum tumour markers for lung cancer have different diagnostic significance for the differential diagnosis of lung cancer pathological types.

**Conclusion:** The quantitative analysis of endobronchial ultrasound elastography combined with tumour markers can improve the diagnosis rate of benign and malignant mediastinal and hilar lymph nodes, help guide the puncture of false negative lymph nodes, and reduce the misdiagnosis rate.

## Introduction

Mediastinal and hilar lymphadenopathy refers to the enlargement of one or more lymph nodes, usually >10 mm in diameter, due to benign or malignant causes, among which lymph node metastasis caused by malignant tumours and granulomatous diseases are the most common [1, 2]. Lung cancer is a malignant tumour with the highest incidence worldwide, accounting for approximately 20% of the mortality rate of all malignant tumours [3]. Its pathological types can be divided into non-small-cell lung cancer (NSCLC) and small-cell lung cancer (SCLC), with NSCLC accounting for approximately 85% of cases. NSCLCs are further divided into lung squamous cell carcinoma (LSCC) and lung adenocarcinoma (LADC) [4]. The 5 year survival rate of lung cancer is 15%–20%. For NSCLC, the 5 year survival rate of stage 1A1 reaches 90%, whereas that of stage IV decreases to <10%. In patients with SCLC, the 5 year survival rate in the limited period is approximately 30%, and that in the extensive period is <10% [5]. The degree of mediastinal and hilar lymph node metastasis caused by lung cancer is a key factor in lymph node staging, which directly affects the tumour-node-metastasis staging results of patients with lung cancer, thus affecting patient prognosis [6, 7]. Therefore, early identification of benign and malignant mediastinal and hilar lymph nodes is critical for clear diagnosis and accurate staging and treatment.

Endobronchial ultrasound-guided transbronchial needle aspiration (EBUS-TBNA) biopsy has become a first-line method for the diagnosis and staging of lymph nodes in lung cancer because of its minimally invasive nature, low complication rate, and high accuracy [8]. With the rapid development of EBUS technology, the application of ultrasonic elastography in the evaluation of lymph nodes has gradually increased.

Elastography provides images of tissue stiffness. According to the different elastic coefficients of different tissues, the degree of tissue deformation after compression by an external force varies. The change in the movement amplitude of the echo signal before and after compression is converted into real-time colour images, which can reflect the characteristic information of diseased tissues [9]. Tissues with a small elastic coefficient and large displacement change after compression are shown in red, tissues with a large elastic coefficient and small displacement change after compression are shown in blue, and tissues with a medium elastic coefficient are shown in green and yellow [10]. Elastography technology, which further expands ultrasound imaging, is a powerful supplement to conventional ultrasonography and can vividly display, locate, and provide information to enable identification of benign and malignant lesions.

A recent meta-analysis found that EBUS elastography has high sensitivity and specificity for the diagnosis of benign and malignant intrathoracic lymph nodes [11]. Intrathoracic lymph nodes can be assessed by qualitative or quantitative methods, such as the strain ratio (SR) and the stiffness area ratio (SAR, blue area in lymph nodes/total area of lymph nodes). Qualitative methods include the elastography type and score [12–17].

Serum tumour markers are important auxiliary markers, having great significance for the screening, diagnosis, and prognosis of lung cancer. Among them, the carcinoembryonic antigen (CEA), cytokeratin-19-fragment (CYFRA21-1), pro-gastrin-releasing peptide (ProGRP), and neuron-specific enolase (NSE) are specific tumour markers for the differential diagnosis of lung cancer and have a high diagnostic sensitivity [18, 19]. However, no previous studies have used on using a combination of elastography and serum lung cancer tumour markers to differentiate between benign and malignant mediastinal and hilar lymph nodes. Therefore, the aim of this study is to combine quantitative analysis of elastic imaging with serum tumour markers, which can improve the accuracy of diagnosis of benign and malignant mediastinum and hilar lymph nodes compared with single diagnosis.

## Materials and methods

### Study design and patients

This study retrospectively analysed patients with mediastinal lymph node enlargement on computed tomography (CT) who underwent EBUS-TBNA at Shengjing Hospital affiliated with the China Medical University from January 2018 to August 2022. All patients were examined for lung cancer serum tumour markers, and elastography was performed before puncture. The serum tumour markers included CEA, CYFRA21-1, ProGRP, and NSE. Patients with incomplete clinical data, such as incomplete detection of tumour markers, absence of elastic imaging images, or poor image quality that could not delineate circular areas were excluded.

### Acquisition and analysis of EBUS examination images

All patients received anaesthesia for EBUS examination, including local airway anaesthetics (lidocaine) or conscious sedatives (midazolam and fentanyl). Elastography was performed using a Pentax EB-1970UK ultrasound bronchoscope and Hitachi HI VISION Avius (Hitachi, Japan) ultrasound scanner.

### Acquisition of the SR value

**FIGURE 1:**
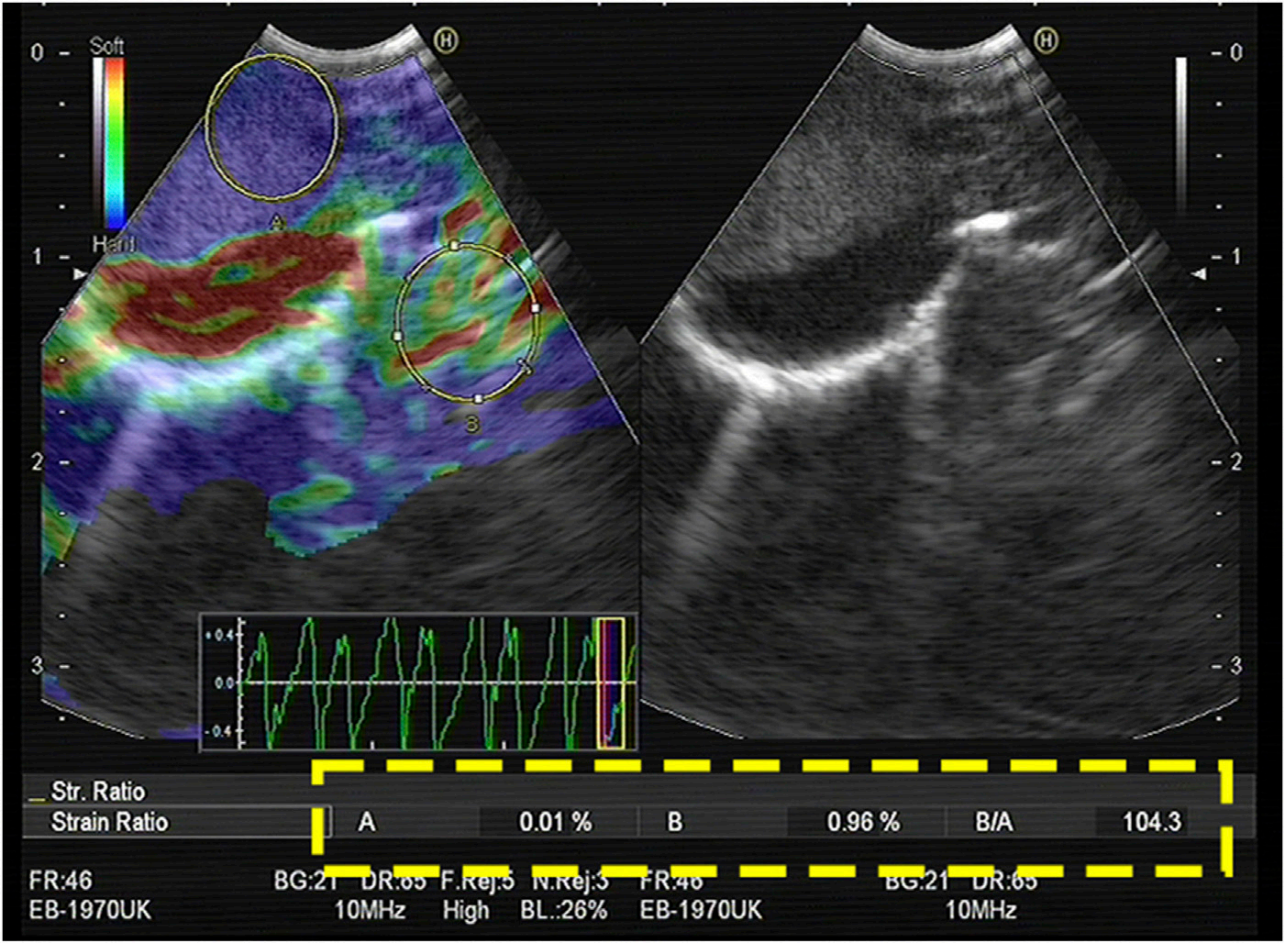
The largest possible area within the LN was outlined as A. An area of the normal-appearing soft tissue outside the LN was selected as B. The strain (B/A) ratio was 104.3. The final diagnosis obtained by EBUS-TBNA was malignant.

The doctor identified the enlarged lymph nodes on preoperative CT images, selected the appropriate lymph nodes for B-mode ultrasound image observation, and switched the imaging system to the elastic imaging mode. After obtaining stable elastic images, the operator applied the functions provided by the equipment to measure the SR of the target lymph nodes in real time. The specific method was as follows: a circle as large as possible was made in the target lymph node, the strain rate of the tissue in the circle was automatically calculated by the device, and the value was recorded as A. Normal tissue was selected to form a circle outside the target lymph node. The circle avoided blood vessels and perivascular tissues in the color Doppler mode, and its size was similar to that of the circle in the target lymph node. Its internal strain rate was denoted as B, and the device automatically calculated the ratio of B to A to calculate the SR (Figure 1). After elastography, each target lymph node was punctured 1–6 times under real-time ultrasound guidance.

### Acquisition of the SAR value

**FIGURE 2:**
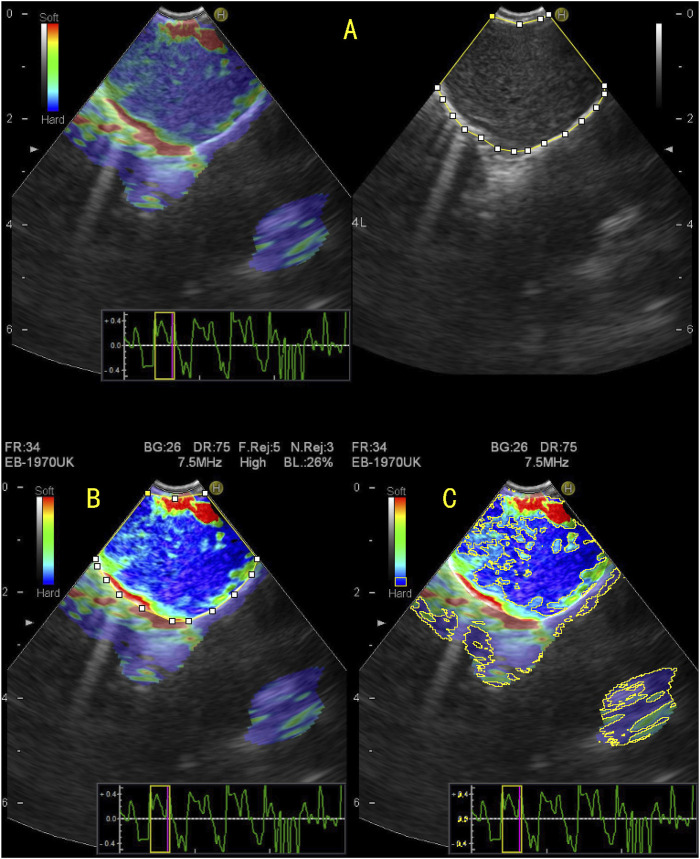
Quantitative evaluation of endobronchial ultrasound elastography using ImageJ. **(A)** The elastography image on the right and the B-mode ultrasound image on the left, on which the entire area of the target lymph node was manually enclosed; **(B)** To enhance the color contrast between the target lymph node and surrounding tissue; **(C)** By adjusting the blue value, brightness, and saturation, the blue region in the target lymph node is displayed and the area of the blue region in the target lymph node is measured. The stiffness area ratio (SAR) was calculated by dividing the blue area (indicating stiffness) by the entire area of the lymph node. SAR = 0.624, pathological diagnosis was malignant.

The elastic image of the target lymph node was retrospectively analysed using the image analysis software ImageJ (Rasband, W.S., ImageJ, U. S. National Institutes of Health, Bethesda, Maryland, United States,^1^ 1997–2018). The blue area and total area of the target lymph node were measured, and the SAR was obtained by calculating the ratio of the two (blue/total). The specific methods were as follows: 1) The picture of the target lymph node to be measured was opened in ImageJ. 2) The “Polygon selections” tool was used to manually outline of the area to be evaluated to calculate the area of the drawn lymph node. 3) The colour contrast between the drawn lymph nodes and the surrounding area was enhanced. 4) The blue area in the lymph nodes was obtained by adjusting the colour value of blue, changing the brightness and saturation, and calculating the blue area. 5) Finally, the SAR was calculated by dividing the resulting blue area by the entire lymph node area (Figure 2).

### Acquisition of the strain rate in lymph nodes (the largest possible area within the LN, LPA) value

The A value was easily and accurately obtained during elastography of the lymph nodes; however, the choice of the B value was often highly subjective and prone to selection bias. Therefore, we studied the A value as an independent parameter and named it the LPA. For the SR and SAR, a value greater than the critical value was considered to indicate malignancy, whereas a value lower than the critical value was considered to indicate benignity. However, as the denominator in the LPA for SR is a small optimal index, values lesser than the critical value were considered to indicate malignancy.

Finally, the pathological results were compared with the results obtained using SR, SAR, and LPA. The final diagnosis of each lymph node was based on pathological and microbiological evidence obtained from EBUS-TBNA, surgery, bronchoalveolar lavage, and bacterial culture, or from a definitive diagnosis obtained at clinical and radiological follow-up (at least 6 months).

### Statistical analysis

Measurement data are expressed as mean ± standard deviation, and the chi-square test was used to compare the data. When the data conditions did not meet the requirements of the chi-square test, the continuous correction chi-square test was used. With pathological diagnosis as the gold standard, a receiver operating characteristic (ROC) curve was constructed to obtain the area under the curve (AUC), sensitivity, specificity, positive predictive value, and negative predictive value of different indicators. The Z-test was used to evaluate whether the differences between the different indicators of the AUC were significant. The Spearman correlation test was used also used to analyse the data. Differences were considered significant when *p* < 0.05. All data were analysed using SPSS 22.0 (IBM Corp., Armonk, NY, United States) and MedCalc 18.0 (MedCalc Software, Ostend, Belgium).

## Results

### Lymph node characteristics

This study enrolled 197 patients, including 109 men and 88 women, aged 17–80 years, with an average age of 57.33 ± 12.14 years. In total, 273 lymph nodes were punctured, with an average of 1.39 lymph nodes per patient. Lymph nodes were classified according to the latest International Staging System [20]. Among the punctured lymph nodes, one was in the 1R group, eight were in the 2R/L group, one was in the 3P group, 64 were in the 4R/L group, 107 were in the 7 group, six were in the 10R/L group, 79 were in the 11R/L group, and seven were in the 12R/L group. Pathological findings showed that 133 lymph nodes were malignant and 140 were benign. Table 1 summarises the patients’ lymph node characteristics.

**TABLE 1:** Characteristics of LNs evaluated in the study.

Characteristic	Numbers of lymph nodes (*N* = 273)	(%)
LN pathology		
Malignant LNs	133	(48.7)
Adenocarcinoma	64	(23.4)
Squamous carcinoma	14	(5.1)
Small cell lung cancer	37	(13.6)
Malignancy of unknown type	11	(4.0)
Lymphoma	4	(1.5)
Metastatic carcinoma	3	(1.1)
Benign LNs	140	(51.3)
LN station		
1R	1	(0.4)
2R/L	8	(2.9)
3P	1	(0.4)
4R/L	64	(23.4)
7	107	(39.2)
10R/L	6	(2.2)
11L	27	(9.9)
11R/Ri/Rs	52	(19.0)
12R/L	7	(2.6)
Average number of punctures	1.39	

### Diagnostic value of quantitative elastography in differentiating benign and malignant mediastinal and hilar lymph nodes

**FIGURE 3:**
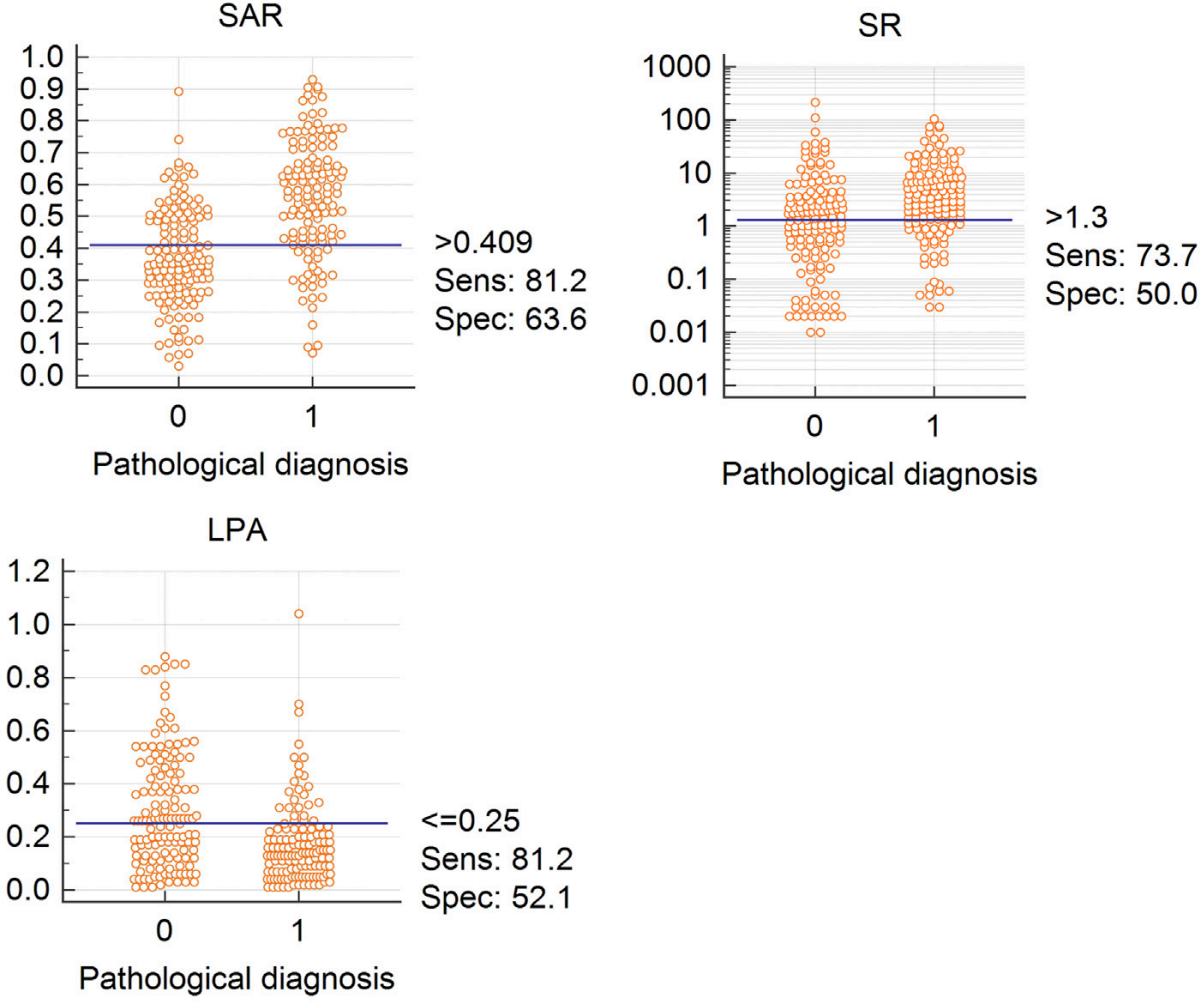
Cutoff values for SAR, LPA, and SR Abbreviation: SAR, stiffness area ratio; SR, strain ratio; LPA, The largest possible area within the LN. “0” is benign, “1” is malignant.

Figure 3 shows the cut-off values of the three quantitative elastography indices in differentiating benign and malignant mediastinal and hilar lymph nodes; the SAR was 0.409, SR was 1.300, and LPA was 0.250, all of which were significant (*p* < 0.05). The diagnostic significance of the SAR was higher than that of the SR (*p* < 0.0001), and the AUC was 0.784 > 0.647. Similarly, the diagnostic significance of the SAR was higher than that of the LPA (*p* = 0.0001), and the AUC was 0.784 > 0.684. There was no significant difference between the diagnostic values of the SR and LPA (*p* = 0.1173). Therefore, the SAR had a higher diagnostic value than the SR and LPA in differentiating benign and malignant mediastinal and hilar lymph nodes. By constructing the ROC curve, the diagnostic rate of the SAR was 72.2%, sensitivity was 81.2%, specificity was 63.6%, and AUC was 0.784. Although the diagnostic rate of the three quantitative elastic imaging combinations was slightly higher than that of the SAR, the difference was not significant (*p* > 0.05) (Table 2).

**TABLE 2:** Diagnostic value of three elastography indexes for benign and malignant lymph nodes.

Characteristic	Cut-off value	Accuracy (%)	Sensitivity (%)	Specificity (%)	PPV (%)	NPV (%)	AUC	*p*-value malignant vs. benign
SAR	0.409	72.2	81.2	63.6	67.9	78.1	0.784	0.000
SR	1.3	61.5	73.7	50.0	58.3	66.7	0.647	0.000
A	0.25	66.3	81.2	52.1	61.7	74.5	0.684	0.000
SAR + A + SR		73.2	79.0	67.9	70.0	77.2	0.796	0.000

Abbreviation: SAR, stiffness area ratio; SR, strain ratio; LPA, The largest possible area within the LN.

### Diagnostic value of serum tumour markers for differentiating benign and malignant mediastinal and hilar lymph nodes

Table 3 summarises the cut-off value and diagnostic value of the four lung cancer tumour markers in differentiating benign and malignant mediastinal and hilar lymph nodes. Compared to single diagnosis, combined diagnosis with CEA, CYFRA21-1, NSE, and ProGRP showed higher specificity and sensitivity, with an AUC of 0.886, which was significant (*p* < 0.05).

**TABLE 3:** Diagnostic value of serum tumor markers for benign and malignant mediastinal and hilar lymph nodes.

Group	Cut-off value	AUC	Sensitivity (%)	Specificity (%)	*p*-value malignant vs. benign
Malignant Tumor					
CEA	4.395 ng/mL	0.801	54.9	92.1	0.000
CYFRA21-1	3.085 ng/mL	0.811	66.9	86.4	0.000
NSE	26.665 ng/mL	0.608	36.1	87.1	0.002
ProGRP	42.99 pg/mL	0.673	62.4	69.3	0.000
Combination of four		0.886	71.4	88.6	0.000

Abbreviation: CEA, carcinoembryonic antigen; CYFRA21-1, cytokeratin-19-fragment; NSE, neuron specific enolase; ProGRP, pro-gastrin-releasing peptide; Combination of four: CEA + CYFRA21-1 + NSE + ProGRP.

### Diagnostic value of quantitative elastography combined with serum lung cancer tumour markers for differentiating benign and malignant mediastinal and hilar lymph nodes

As shown in Table 4, among the three quantitative elastography indices and tumour markers, only the SAR was positively correlated with the CEA, CYFRA21-1, and NSE. Although the correlation was not high, there was a significant difference between them (*p* < 0.05). The SAR combined with CEA, CYFRA21-1, and NSE had a high diagnostic significance for mediastinal and hilar lymph nodes, with a sensitivity of 79.7%, specificity of 90.7%, positive predictive value of 89.1%, and negative predictive value of 82.5%. The AUC was 0.926, which had a higher diagnostic value than that of tumour markers or quantitative elastography alone (*p* < 0.05) (Table 5).

**TABLE 4:** Correlation of SAR and serum tumor markers (CEA, CYFRA21-1, NSE, ProGRP).

Relative factors	CEA	CYFRA21-1	NSE	ProGRP
SAR	0.202**	0.162**	0.123*	0.088
LPA	−0.066	−0.076	−0.088	−0.057
SR	0.027	−0.018	0.044	−0.027

Note: * and ** mean significant correlation at *p* < 0.05 and *p* < 0.01.

Abbreviation: SAR, stiffness area ratio; SR, strain ratio; LPA, The largest possible area within the LN; CEA, carcinoembryonic antigen; CYFRA21-1, cytokeratin-19-fragment; NSE, neuron specific enolase; ProGRP, pro-gastrin-releasing peptide.

**TABLE 5:** The diagnostic value of SAR combined with serum tumor markers in mediastinal and hilar lymph nodes.

Characteristic	Accuracy (%)	Sensitivity (%)	Specificity (%)	PPV (%)	NPV (%)	AUC
SAR + CEA	80.2	83.5	77.1	77.6	83.1	0.858
SAR + CYFRA21-1	82.4	80.5	84.5	82.9	81.9	0.878
SAR + NSE	76.2	72.9	79.3	77.0	75.5	0.819
SAR + ProGRP	73.6	66.9	80.0	76.1	71.8	0.791
SAR + CEA + CYFRA21-1	84.2	88.0	80.7	81.2	87.6	0.907
SAR + CEA + CYFRA21-1 + NSE	85.4	79.7	90.7	89.1	82.5	0.926

Abbreviation: SAR, stiffness area ratio; CEA, carcinoembryonic antigen; CYFRA21-1, cytokeratin-19-fragment; NSE, neuron specific enolase; ProGRP, pro-gastrin-releasing peptide.

Table 6 shows the diagnostic value of the four tumour markers combined with different quantitative analysis indices in elastography. Compared to those of the SAR combined with the CEA, CYFRA21-1, and NSE, the diagnostic rate and sensitivity of the SAR combined with the four serum lung cancer tumour markers were 85.7% and 83.5%, respectively, with an AUC of 0.927. The combination of the four serum lung cancer tumour markers and the three quantitative elastography indicators had the highest diagnostic value, with a diagnostic rate of 86.4%, a sensitivity of 83.5%, a specificity of 89.3%, and an AUC of 0.930.

**TABLE 6:** Comparison of diagnostic value for combined characteristics in benign/malignant differentiation.

Combined characteristic	Accuracy (%)	Sensitivity (%)	Specificity (%)	PPV (%)	NPV (%)	AUC
SAR + tumor markers	85.7	83.5	87.9	86.7	84.8	0.927
A+ tumor markers	82.8	88.7	77.1	78.7	87.8	0.905
SR + tumor markers	80.2	71.4	87.9	85.8	76.4	0.889
SAR + A+ tumor markers	85.7	83.5	87.9	86.7	84.8	0.927
SAR + A+ SR + tumor markers	86.4	83.5	89.3	88.1	85.0	0.930

Abbreviation: SAR, stiffness area ratio; SR, strain ratio; LPA, The largest possible area within the LN; tumor markers, CEA + CYFRA21-1 + NSE + ProGRP.

### Diagnostic value of quantitative elastography in pathological types of lung cancer

**FIGURE 4:**
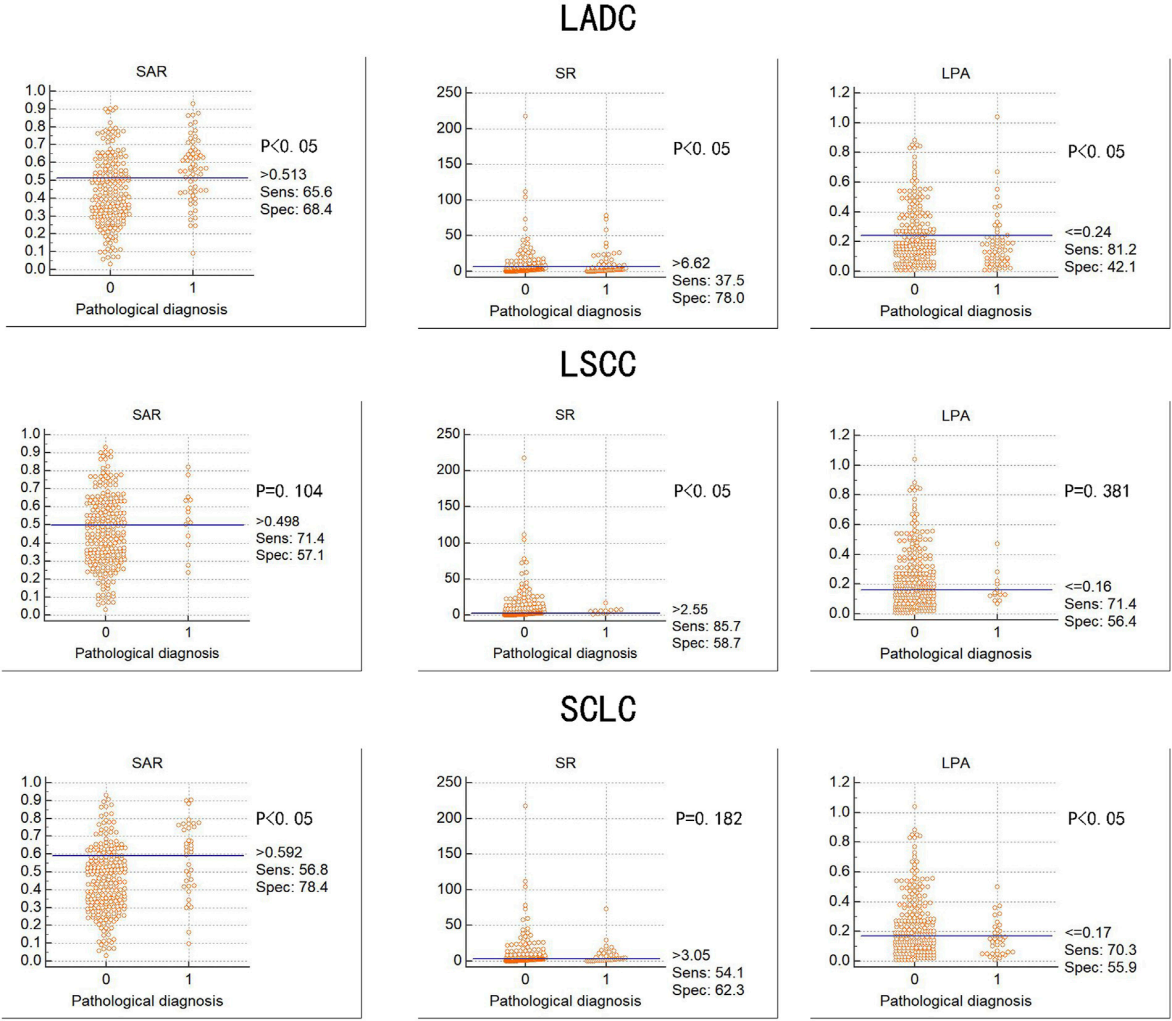
Diagnostic value of three elastography parameters for different types of lung cancer Abbreviation: SAR, stiffness area ratio; SR, strain ratio; LPA, The largest possible area within the LN. LADC, Lung adenocarcinoma; LSCC, Lung squamous cell carcinoma; SCLC, Small cell lung cancer. “0” is benign, “1” is malignant.

Figure 4 summarises the diagnostic value of the three quantitative elastography indicators for LADC, LSCC, and SCLC. In LADC, the cut-off values of the SAR, SR, and LPA were 0.513, 6.620, and 0.240, respectively, which were significant. In LSCC, only the SR was significant, with a cut-off value of 2.550; in SCLC, the SAR and LPA were significant, with cut-off values of 0.592 and 3.050, respectively. Compared with the SR and LPA, the SAR had the highest diagnostic value for LADC and SCLC (*p* < 0.05).

### Diagnostic value of serum lung cancer tumour markers for pathological types of lung cancer

Table 7 summarises the cut-off values, sensitivity, and specificity of the four serum lung cancer tumour markers for LADC, LSCC, and SCLC. Among them, the combined detection rate of the CEA and CYFRA21-1 for LADC and NSE and ProGRP for SCLC and single detection rate of the CYFRA21-1 for LSCC had a high diagnostic significance.

**TABLE 7:** Diagnostic efficiencies of four serum tumor markers for lung cancers of different pathological types.

Group	Cut-off value	AUC	Sensitivity (%)	Specificity (%)	*p*-value malignant vs. benign
Malignant Tumor					
CEA	4.395 ng/mL	0.801	54.9	92.1	0.000
CYFRA21-1	3.085 ng/mL	0.811	66.9	86.4	0.000
NSE	26.665 ng/mL	0.608	36.1	87.1	0.002
ProGRP	42.99 pg/mL	0.673	62.4	69.3	0.000
Combination of four		0.886	71.4	88.6	0.000
LADC					
CEA	4.605 ng/mL	0.808	68.8	85.6	0.000
CYFRA21-1	3.145 ng/mL	0.784	78.1	73.2	0.000
NSE	13.995 ng/mL	0.489	89.1	18.2	0.792
ProGRP	20.74 pg/mL	0.449	100	5.7	0.218
CEA + CYFRA21-1		0.870	82.8	80.9	0.000
Combination of four		0.807	78.1	71.3	0.000
SCLC					
CEA	2.03 ng/mL	0.629	85.5	44.9	0.012
CYFRA21-1	2.06 ng/mL	0.534	83.8	39	0.509
NSE	30.155 ng/mL	0.832	70.3	90.3	0.000
ProGRP	97.995 pg/mL	0.894	81.1	97.0	0.000
NSE + ProGRP		0.914	94.6	74.6	0.000
Combination of four		0.747	73.0	72.5	0.000
LSCC					
CEA	1.32 ng/mL	0.563	100	23.2	0.424
CYFRA21-1	3.855 ng/mL	0.836	78.6	73.0	0.000
NSE	17.77 ng/mL	0.437	71.4	41.3	0.428
ProGRP	30.24 pg/mL	0.504	100	21.6	0.961
Combination of four		0.697	64.3	71.8	0.013

Abbreviation: CEA, carcinoembryonic antigen; CYFRA21-1, cytokeratin-19-fragment; NSE, neuron specific enolase; ProGRP, pro-gastrin-releasing peptide; LADC, lung adenocarcinoma; LSCC, lung squamous cell carcinoma; SCLC, small cell lung cancer; Combination of four: CEA + CYFRA21-1 + NSE + ProGRP.

### Diagnostic value of quantitative elastography indicators combined with serum lung cancer tumour markers for pathological types of lung cancer

Table 8 shows the diagnostic value of quantitative elastography indicators combined with tumour markers specific to lung cancer types, including LADC, LSCC, and SCLC. In LADC, the AUC of the SAR, SR, and LPA combined with the CEA and CYFRA21-1 was 0.866. In SCLC, the AUC of the SR and LPA combined with the NSE and ProGRP was 0.889. The diagnostic rate and specificity of the three quantitative elastography indices combined with the NSE and ProGRP were the highest at 92.3% and 95.3%, respectively, and the AUC was 0.896. In LSCC, the diagnostic rate of the SR combined with the CYFRA21-1 was 78.8%, and the AUC was 0.835; the sensitivity of the SAR, SR, and LPA combined with the CYFRA21-1 was 1, and the AUC was 0.839. There was no significant difference in the diagnostic value of the three quantitative elastography indicators combined with tumour markers than that of tumour markers alone for lung cancer types (*p* > 0.05).

**TABLE 8:** Diagnostic efficiency of elastography combined with tumor markers for different types of lung cancer.

Parameters	Accuracy (%)	Sensitivity (%)	Specificity (%)	PPV (%)	NPV (%)	AUC	*p*-value malignant vs. benign
LADC							
SAR	67.8	65.6	68.4	38.9	86.7	0.696	0.000
SAR + CEA + CYFRA21-1	77	84.4	74.6	50.5	94	0.864	0.000
a + CEA + CYFRA21-1	78.7	81.3	78	53.1	93.1	0.866	0.000
CEA + CYFRA21-1	83.4	82.8	80.9	57	93.9	0.870	0.000
SCLC							
SAR + NSE + ProGRP	84	83.8	83.9	44.9	97.1	0.891	0.000
SAR + LPA + NSE + ProGRP	84	83.8	83.9	44.9	97.1	0.889	0.000
a + NSE + ProGRP	92.3	73	95.3	71.1	95.7	0.896	0.000
NSE + ProGRP	77.6	94.6	74.6	36.8	98.9	0.914	0.000
LSCC							
SR	71.2	85.7	58.7	10.1	98.7	0.677	0.000
SR + CYFRA21-1	78.8	71.4	79.2	15.6	98.1	0.835	0.000
a + CYFRA21-1	58.9	100	56.8	11.1	100	0.839	0.000
CYFRA21-1	72.8	78.6	73	13.6	98.4	0.836	0.000

Abbreviation: SAR, stiffness area ratio; SR, strain ratio; LPA, The largest possible area within the LN; a, SAR + SR + A.

## Discussion

This is the first time the diagnostic value of quantitative elastography combined with serum lung cancer tumour markers for benign and malignant mediastinal and hilar lymph nodes has been analysed in detail. The results show that quantitative elastography and serum tumour markers have diagnostic significance for benign and malignant mediastinal and hilar lymph nodes, and their combination significantly further improves diagnosis.

Mediastinal and hilar lymphadenopathy are commonly caused by malignant tumours, such as lung cancer, and granulomatous diseases. As lung cancer has the highest incidence worldwide and a high rate of lymph node metastasis, accurate lymph node staging is particularly important for treatment planning. Mediastinoscopy is considered the gold standard for the diagnosis of mediastinal lymph node metastasis; however, with the continuous development of endoscopic ultrasonography, EBUS-TBNA has gradually become the first-line method for the diagnosis and staging of mediastinal lymph nodes [8]. A study comparing the significance of EBUS examination and mediastinoscopy in lung cancer staging showed no significant difference in the sensitivity, positive predictive value, and accuracy of the two for diagnosing malignant lymph nodes [21].

Conventional ultrasonography has advantages over mediastinoscopy, due to real-time visualization, and being dynamic and less invasive. During EBUS-TBNA, the nature of the lymph nodes can be preliminarily determined according to the ultrasound characteristics. Fujiwara et al. [22] conducted a retrospective study of 1,061 cases of mediastinal and hilar lymph nodes and found that round, clear boundaries, uneven internal echoes and the presence of coagulation necrosis can be used as independent predictors for the diagnosis of malignant lymph nodes. However, there are large differences in the evaluation of B-mode ultrasonography of lymph nodes, and simultaneously, the operator requires sufficient experience [13, 16, 22, 23].

As a recent, non-invasive diagnostic technology, ultrasound elastography has a high diagnostic rate and the results are easy to evaluate [17, 24]. Lymph node evaluation using elastic imaging can be divided into qualitative and quantitative evaluations [25]. In a qualitative assessment, Izumo et al. [12] confirmed that elastic image types have a certain value in distinguishing benign and malignant thoracic lymph nodes. Further, the elastic score of malignant lymph nodes was significantly higher than that of benign lymph nodes [26]. However, the classification of image types is subjective, and the definition and classification of intermediate types of lymph nodes (some blue and some non-blue) are unclear. A quantitative assessment can partially eliminate this uncertainty using specific numerical values. Therefore, this study focused on a quantitative assessment of elastography.

In a quantitative assessment, Korrungruang and Boonsarngsuk [27] found that the SR of the malignant group was significantly higher than that of the benign group. The authors suggest that the use of the SR to distinguish benign and malignant lymph nodes has a high diagnostic value and that the SR is the only quantitative method that can evaluate lymph nodes and surrounding tissues in real time. Rozman et al. [13] also concluded that the SR could more accurately distinguish between malignant and benign lymph nodes. Because there are subjective differences in classifying the colour distribution in elastography, it is particularly important to calculate the ratio of the blue area within the lymph node to the total area of the lymph node. Ma et al. [15] confirmed that the ratio of the blue area to the total area of lymph nodes is of great significance in differentiating benign and malignant mediastinal and hilar lymph nodes. One study demonstrated that evaluating the SAR of lymph nodes before puncture can help guide the puncture and increase the diagnostic rate [28]. Additionally, Uchimura et al. [17] found that the SAR also had a high diagnostic significance for malignant tumours with a short mediastinal lymph node having a diameter <10 mm.

In the measurement of the SR, the LPA was previously used to directly reflect the strain rate in the target lymph node [27], which excluded the selection bias of normal tissue. Herein, we analysed the diagnostic value of the LPA for the first time, and the results showed that the LPA could be used for the differential diagnosis of benign and malignant mediastinal and hilar lymph nodes. The cut-off value was 0.25, sensitivity was 81.2%, and specificity was 52.1%, all of which were better than those of the SR. Although the difference was not significant (χ^2^ = 3.12, *p* = 0.0773; χ^2^ = 0.13, *p* = 0.718), its advantages could not be ignored.

In this study, we used three quantitative elastography parameters to study: the SAR, SR, and LPA. We found that the SAR had the greatest significance in the diagnosis of benign and malignant mediastinum and hilar lymph nodes.

Quantitative elastography reflects the degree of tumour stiffness and distinguishes benign and malignant tissues; however, changes in tumour stiffness can cause changes in the tumour microenvironment, which is specifically reflected in the expression of serum tumour markers [29]. Therefore, we analysed the correlation between the SAR, SR, LPA, and serum tumour markers in lung cancer. Incidentally, we found that there was a positive correlation between the SAR and CEA, CYFRA21-1, and NSE. Although the correlation was not high, the combination of the SAR with the CEA, CYFRA21-1, and NSE significantly improved the diagnostic value for benign and malignant lymph nodes. Hao et al. [29] studied the diagnostic value of elastic imaging SR and serum tumour markers for breast cancer and found a correlation between the SR and tumour marker cancer antigen 15-3 (CA15-3), and their combination significantly improved the differential diagnosis of benign and malignant tumours. This finding reflects the importance of quantitative elastography combined with serum tumour markers in differentiating benign from malignant tumours. Therefore, we combined the three elastography indices with serum tumour markers and found that the combined diagnostic model improved the diagnostic value for benign and malignant mediastinal and hilar lymph nodes, with an AUC of 0.930, a diagnostic rate of 86.4%, a sensitivity of 83.5%, and a specificity of 89.3%. However, these are all auxiliary clinical methods for the diagnosis of benign and malignant tumours, and the pathological results should be considered as the gold standard.

Herein, the cut-off value of the SAR obtained was similar to that reported in a recent study (0.412) [17], but there were some indicators with lower cut-off values of 0.311 and 0.367 [15, 28], which might be due to differences in the characteristics and number of enrolled patients. The cut-off value of the SR was 1.3, which was different from the values of 2.5 and 8.0 previously reported [13, 27]. This may be because central necrosis and vascular infiltration usually occur in lymph nodes, resulting in reduction of the SR due to “softening” in the lymph nodes [30], different criteria for image selection (for example, the optimal ratio of the SR is recommended to be lesion/normal tissue = 1:1 [31]), and the influence of the operators and equipment.

Recently, many non-invasive screening studies have been conducted on the expression of various serum lung cancer tumour markers. There are also many reports with different views on the diagnostic value of serum lung cancer tumour markers for different types of lung cancers [19, 32–34]. In this study, we found that the CYFRA21-1 combined with the CEA was helpful in diagnosing LADC; the ProGRP and NSE were helpful in diagnosing SCLC; and the CYFRA21-1 was helpful in diagnosing LSCC, similar to the results of previous studies [33, 34]. Nevertheless, a study has shown that the CYFRA21-1 combined with the squamous cell carcinoma antigen is more helpful in the diagnosis of LSCC [34]. Therefore, the selection of effective combined detection plays an important role in the early detection of the disease and is worthy of clinical promotion and application. Additionally, we found that quantitative elastography indicators also had diagnostic significance for LADC, LSCC, and SCLC, and the SAR had the highest diagnostic value for LADC and SCLC, with cut-off values of 0.513 and 0.592, respectively. However, the SAR of LSCC was not significant; only the SR was significant, with a cut-off value of 2.55. One study found that the average SAR of LSCC was 0.716 [15]. This may be because of the low number of confirmed LSCCs.

To further improve the diagnosis of pathological types of lung cancer, we combined quantitative elastography indicators with tumour markers, but the results were not as expected. Compared with tumour markers for pathological types of lung cancer, the diagnostic value of quantitative elastography combined with tumour markers for pathological types of lung cancer was not significantly improved, and there was no statistical significance between the two (*p* > 0.05), and even in LADC and SCLC, its diagnostic value was reduced. The reason for this may be related to the relatively poor hardness of SCLC and LADC and the small number of pathological types of lung cancer.

In our study, the SAR on elastography showed great significance in differentiating between benign and malignant mediastinal and hilar lymph nodes. However, to evaluate the SAR of a lymph node, it is necessary to download the elastic image from the ultrasound machine to the computer and analyse the static elastic image using software. It takes approximately 1 min to do this, which increases the labour and time consumption. With the application of artificial intelligence in elastography, our work efficiency is greatly improved, and selection bias caused by static image analysis is avoided [35].

This study has several limitations. First, it was a retrospective study. Second, only 1–3 respiratory physicians assessed the elastography results and determined the final diagnosis. To some extent, there may be operator selection bias, but this is an inevitable drawback in most elastography studies. Finally, the elastic images selected for the SAR calculation were static; therefore, bias in image selection may also have occurred.

Clinically, patients with a history of dust exposure and central necrotic lymph nodes may increase the blue area in the lymph nodes, thus affecting the results of elastic imaging. Meanwhile, tumor markers are also increased under the influence of inflammation and other factors. Therefore, in this paper, we emphasize the combined diagnosis of the two indicators, which can greatly reduce the influence of these factors. Improve our rate of diagnosis of malignancy and puncture success rate.

In the future, we should pay attention to the results of serum tumour markers before puncture, carefully analyse the results of CT images and ultrasound features of lymph nodes, conduct real-time elastic imaging analysis, and select the exact puncture site using SAR results to maximise the diagnostic rate. Further prospective multicentre trials are required to confirm this study’s results.

## Conclusion

In summary, our findings suggest that the combination of quantitative elastic imaging and serum tumour markers has a higher diagnostic value than the detection of tumour markers and elastography alone in differentiating benign and malignant mediastinal and hilar lymph nodes, and it may help guide the puncture of false-negative lymph nodes.

## Data Availability

The original contributions presented in the study are included in the article/supplementary material, further inquiries can be directed to the corresponding author.

## Footnotes

1
https://imagej.nih.gov/ij/
